# Assessing Predictive Factors of Attitudes Toward Peer-Supported Mental Health Interventions in the Metaverse: Mixed Methods Study

**DOI:** 10.2196/57990

**Published:** 2024-08-22

**Authors:** Francisco Nicolas Ramos, Rachel A Bernstein, Iony D Ezawa

**Affiliations:** 1 Department of Psychology University of Southern California Los Angeles, CA United States

**Keywords:** metaverse, mental health intervention, consumer attitude, digital mental health intervention, virtual world

## Abstract

**Background:**

The metaverse is a promising avenue for accessible, effective digital mental health treatments. However, general attitudes toward peer-supported metaverse mental health interventions (MMHIs) remain largely unexplored.

**Objective:**

This study examined the relation of sociodemographic, mental health, and technology factors in predicting attitudes toward MMHIs.

**Methods:**

We used a mixed methods design with a self-report online survey (N=545 participants) to assess participant attitudes toward MMHIs and sociodemographic, mental health, and technology factors. Ordinal logistic regression was used to examine predictors of general interest in peer-supported MMHIs and binary logistic regression to examine predictors of preference for MMHIs versus face-to-face interventions. Inductive content analysis was performed on 483 open-ended responses regarding intervention preference.

**Results:**

Older age (odds ratio [OR] 1.03, 95% CI 1.02-1.05; *P*<.001), higher ethnic identity centrality (OR 1.44, 95% CI 1.25-1.66; *P*<.001), more positive mental help–seeking attitudes (OR 1.22, 95% CI 1.06-1.42; *P*=.007), more online video game use (OR 1.26, 95% CI 1.09-1.44; *P*=.001), and greater virtual reality experience (OR 1.55, 95% CI 1.28-1.90; *P*<.001) were associated with greater odds of reporting more interest in MMHIs. Internet access was associated with greater odds of reporting less interest in MMHIs (OR 0.50, 95% CI 0.30-0.84; *P*=.01). Hispanic ethnicity (OR 1.81, 95% CI 1.13-2.90; *P*=.01), older age (OR 1.04, 95% CI 1.02-1.05; *P*<.001), higher ethnic identity centrality (OR 1.28, 95% CI 1.09-1.51; *P*=.003), smartphone access (OR 10.46, 95% CI 2.87-50.71; *P*<.001), higher self-reported video game use (OR 1.25, 95% CI 1.05-1.48; *P*=.01), and more positive computer attitudes (OR 1.05, 95% CI 1.01-1.10; *P*=.02) predicted greater odds of preference for MMHIs (versus face-to-face interventions), whereas the male gender (OR 0.43, 95% CI 0.28-0.68; *P*<.001), internet access (OR 0.12, 95% CI 0.02-0.40; *P*=.002), more positive mental help–seeking attitudes (OR 0.76, 95% CI 0.62-0.92; *P*=.005), and moderately severe (OR 0.20, 95% CI 0.07-0.51; *P*=.001) and severe (OR 0.26, 95% CI 0.08-0.79; *P*=.02) levels of depression symptoms predicted lower odds of preference for MMHIs. Qualitative analysis revealed 14 themes describing reasons for intervention preference. Anonymity (133/483, 27.5%), social aversion (38/483, 7.9%), ease of use and accessibility (35/483, 7.2%), anxiety (28/483, 5.8%), and comfort (26/483, 5.4%) tended to be endorsed by those preferring MMHIs. Ecological validity of social interactions (99/483, 20.5%), ecological validity of interventions (75/483, 15.5%), aversion/distrust toward technology (42/483, 8.7%), impersonal quality (31/483, 6.4%), and immersion/engagement (11/483, 2.3%) tended to be endorsed by those who preferred face-to-face interventions. Mental health attitudes (28/483, 5.8%), privacy (19/483, 3.9%), and miscellaneous reasons (46/483, 9.5%) were endorsed equally between preferences. Novelty (21/483, 4.3%) was most cited by those who expressed no preference.

**Conclusions:**

This study identified several factors associated with attitudes toward peer-supported MMHIs, which may be leveraged to inform mental health outreach to interested populations.

## Introduction

### Background

In efforts to connect mental health services with those in need, novel technologies are increasingly used to facilitate mental health treatment. Recently, there has been significant development in the technology of the metaverse and its implications for the future of health care [1,2]. The metaverse, a collection of enduring, interoperable 3D virtual worlds in which people interact with each other and the environment using virtual avatars [3], shows promise as a platform for mental health interventions for several reasons. First, metaverse mental health interventions (MMHIs) are situated *between* technology-based interventions (which use technology to create unique therapeutic affordances) and mere technology-facilitated interventions (eg, psychotherapy delivered by video calls) [4]. This is because MMHIs use customizable, anonymous avatars to mediate social communication, which may appeal to individuals who would otherwise not seek treatment due to stigma, anxiety, or fear of discrimination in face-to-face therapy [5-8]. Second, MMHIs share many of the advantages of the wider umbrella of digital mental health interventions (DMHIs), including a controlled environment with integrated data collection and accessibility from a range of devices and locations [9]. Third, the social aspect of the metaverse offers simple integration of peer support programs [8]. Research suggests online peer support to be associated with improved depression symptomatology [10,11] and general peer support with increased engagement and efficacy in internet therapies [12].

The investigation of attitudes toward peer-supported MMHIs is critical to their development because it will aid in the identification of populations for whom MMHIs may facilitate mental help–seeking, as well as of barriers to seeking out these types of interventions. We are not aware of any research assessing factors that may influence attitudes toward MMHIs, but the broader literature on DMHIs suggests several factors that may have a significant impact on attitudes toward MMHIs.

### Sociodemographic Factors

Research assessing the associations between common sociodemographic factors and attitudes toward DMHIs presents mixed results. For example, previous studies evaluating attitudes toward DMHIs report conflicting results as to whether associations with gender or age exist, as well as the direction of the associations found [13-17]. However, because the umbrella of interventions that can be defined as DMHIs is broad, these studies assess different interventions, from online peer support groups [13] to online psychotherapy delivered by a professional [17]. The investigation of attitudes toward peer-supported MMHIs is narrow in scope, which may aid in accuracy in identifying the relationships between relevant sociodemographic factors and attitudes toward peer-supported MMHIs. In addition, several key sociodemographic factors that could be associated with attitudes toward MMHIs have not been previously evaluated in the DMHI literature. Technological access variables, such as internet access, are negatively associated with self-reported poor mental health [18] and could have a relationship with how one perceives the accessibility, pricing, or usability of technology-based MMHIs. Lastly, ethnic identity centrality (the importance one’s ethnicity has to their identity as a whole) could play a role in attitudes toward MMHIs due to the use of customizable avatars that participants can use to either hide or highlight parts of their identities. It is critical to assess the sociodemographic factors that correlate with attitudes toward MMHIs in order to understand which populations may be particularly willing or unwilling to use MMHIs.

### Mental Health Factors

Synchronous DMHIs may be preferred to face-to-face interventions for individuals experiencing common mental health disorders, such as anxiety (particularly social anxiety) and depression, due to reduced social interaction–related stress [19] and reduced stigma and embarrassment when the DMHI is anonymous [20]. MMHIs are synchronous and anonymous, which may make them particularly attractive to individuals with elevated levels of social anxiety or depression symptoms. Furthermore, negative attitudes toward mental help–seeking remain a barrier to the use of DMHIs [20]. The unique features of MMHIs, such as their ability to balance a sense of presence and anonymity [7], may prove attractive to those who would otherwise not seek treatment due to negative attitudes toward mental help–seeking.

### Technology Factors

Attitudes toward technology appear to be generally related to attitudes toward DMHIs [21]. Since the metaverse and virtual worlds are historically computer based [22], attitudes toward computers specifically may be related to attitudes toward peer-supported MMHIs. Moreover, peer-supported MMHIs share strong mechanistic similarities with online multiplayer video games. Not only do such games facilitate anonymous communication and group social interaction, but most of the mechanics and navigation elements of MMHIs would be familiar to gamers, such as “avatars,” “push to talk” buttons, and immersion in a virtual world. Online video game use may, therefore, be a pertinent factor in attitudes toward peer-supported MMHIs.

### Study Aims

The aim of this study was to assess factors that may predict general interest in MMHIs, as well as preference for MMHIs versus face-to-face interventions. To accomplish this objective, we conducted a web-based survey in a diverse sample from the general population to assess sociodemographic, mental health, and technology factors that could be associated with attitudes toward peer-supported MMHIs.

## Methods

### Study Design

This study involved a mixed methods design with data gathered from an open, voluntary, web-based survey. The online survey included a variety of quantitative self-report measures of attitudes toward different interventions, sociodemographic factors, mental health factors, and technology factors. The survey also included open-ended questions used to gather qualitative data on participants’ reasons for their attitudes toward intervention types. We chose this study design because it is feasible for participants and can easily reach a large, diverse sample. Reporting followed the Checklist for Reporting Results of Internet E-Surveys (CHERRIES; see Multimedia Appendix 1 for the checklist) [23].

### Recruitment

Inclusion criteria for the study were (1) ability and willingness to provide informed consent and (2) age of 18 years or above. There were no exclusion criteria for this study. Participants meeting these criteria were recruited to participate in the survey using word-of-mouth, flyers distributed around our university campus, social media posts on the networks Reddit and X (formerly Twitter), and Prolific (a web-based recruitment platform) [24]. Recruitment occurred from April to July 2023. Initial contacts for recruitment occurred online via the social media posts, except for a few individuals who received a paper flyer on our university campus. For social media, a single post was made to each social media site with information about the study and a direct link to complete the study consent form and survey. The survey was posted 4 times on Prolific to target different individuals identifying as part of various racial/ethnic groups (using Prolific’s available demographic-screening options).

We calculated our anticipated sample size using the recommendation of at least 10 events per variable in a logistic regression [25-27]. This recommendation helps avoid overfitting and biased estimates. We originally planned to include 19 predictors in our model, which resulted in an estimated sample size of 380 participants. However, some of these predictors were removed after preliminary examination of the data (eg, presence of highly related predictors). This recommendation is only a minimum, and a higher number of events per variable is almost always preferable [28]. Due to the unexpected strong success of our recruitment strategies, recruitment continued until we could no longer fund recruitment.

### Procedures

Participants who accessed the study link via the online posts or by scanning the QR code on paper flyers were redirected to a University of Southern California (USC) Qualtrics page and presented with an information sheet for the study. The information sheet included the estimated study length of 9 minutes and a data storage policy for the collection of anonymous data. Participants were then asked to provide consent. Those who consented to participate in the study were asked to complete a series of self-report measures assessing sociodemographic, mental health, and technology factors. Participants were then asked to watch a short video clip showing an example of a mental health intervention taking place in the metaverse (Innerworld, developed by Innerworld, Inc [7], was used as an example of a peer-supported MMHI). Lastly, participants completed measures of their attitudes toward MMHIs. Prolific participants were compensated with US $2 for completing the survey, while all other participants were provided with the chance to be selected for a US $100 gift card raffle.

The survey was hosted on Qualtrics with a black-and-white color scheme and official USC branding. We implemented IP address monitoring and browser cookie measures in Qualtrics in order to prevent the same user from completing the survey multiple times during the duration of the study. The survey’s usability and functionality were assessed by investigators and close colleagues prior to data collection. An attention check question was included, which participants had to answer correctly to proceed with the survey. The survey used adaptive questioning to reduce participant burden, but we did not randomize the order of items or question blocks for individual participants. Respondents could not review previous answers. Completeness was reported automatically by Qualtrics, but survey responses were also reviewed manually. Fraudulent responses were removed after a thorough examination, including checking for impossible or exceptionally fast time stamps (at least 3 SDs below the mean duration), abnormally low clicks counted, nonsensical or artificial intelligence (AI)-generated open-ended responses, and “straight-lined” answers.

### Ethical Considerations

This study was approved by the USC’s Institutional Review Board at the exempt level (approval number: UP-23-00491). Data were anonymized and contained no identifying information.

### Measures

#### Sociodemographic Factors

Participants were asked to provide their ethnicity/race, gender identity, and age. Participants were also asked whether they had access to the internet or a smartphone or both. Consistent with prior work on ethnic identity centrality [29-31], we asked participants to indicate their level of ethnic identity centrality (“How important is your racial or ethnic background to your identity as a whole?”) on a 5-point scale ranging from 1 (not at all important) to 5 (extremely important).

#### Mental Health Factors

##### Mental Help–Seeking Attitudes

The Mental Help Seeking Attitudes Scale (MHSAS), a 9-item self-report measure, was used to evaluate participant attitudes toward seeking help from a mental health professional. The MHSAS is a validated [32] bipolar scale with 7 response options to each item anchored on either end by dichotomous adjectives (eg, good, bad). A mean total score is computed, with higher scores indicating more positive attitudes toward seeking mental help.

##### Level of Depression Symptoms

Depression symptoms were measured using the 8-item Patient Health Questionnaire (PHQ-8), a well-validated and reliable self-report measure of depression symptoms [33]. Each item is rated on a 0–3-point Likert scale, and a sum total score is computed, with higher scores indicating greater depression symptom severity. This variable was categorized according to severity categories set by the authors of this instrument: 0-4 indicating no symptoms, 5-9 indicating mild symptoms, 10-14 indicating moderate symptoms, 15-19 indicating moderately severe symptoms, and ≥20 indicating severe symptoms.

##### Level of Social Anxiety Symptoms

Social anxiety symptoms were measured using the Mini-Social Phobia Inventory (Mini-SPIN) [34]. The Mini-SPIN is a validated [35-37] 3-item self-report screening tool that asks participants to rate, in the past week, how much (ranging from 0 for *not at all* to 4 for *extremely*) they have been bothered by problems related to social anxiety. A sum total score is computed, with higher scores indicating greater social anxiety symptom severity. This variable was dichotomized based on suggestions from the authors of this instrument, with a score of 6 or greater indicating the presence of a clinical level of symptom severity.

#### Technology Factors

##### Computer Attitudes

To evaluate attitudes toward using computers, we adapted the Computer Attitudes subscale of the well-validated Computer Aversion, Attitudes, and Familiarity Index (CAAFI) [38-40]. The subscale consists of 9 items asking participants to select the response, ranging from –3 (absolutely false) to 3 (absolutely true), that best describes how true or false a statement is to them. A sum total score is computed, with higher scores indicating more positive attitudes toward using computers. We removed 3 items that were outdated with regard to the cultural mass usage of computers and email in the recent decade (“I use a computer input device every day,” “I use email every day,” and “Email is an easy way to communicate with people.”).

##### Video Game/Internet Use Habits

Similarly to previous research assessing internet habits [41,42], participants self-reported how often (ranging from 1 for *never* to 5 for *very often*) they engaged in specific activities while connected to the internet (eg, “playing online video games”).

##### Virtual Reality Experience

Consistent with prior work [43,44], we used a single item with 4 response options ranging from 1 (no experience) to 4 (a lot of experience) to assess participants’ previous experience with virtual reality (VR) technology.

#### Outcome Variables

##### General Interest in Peer-Supported MMHIs

We adapted a single-item measure commonly used across disciplines to assess overall willingness to use a peer-supported MMHI [45,46]. Participants were asked how willing they would be to use a peer-supported MMHI if money was not a concern, with response options ranging from 1 (not at all willing) to 5 (extremely willing).

##### Preference for Peer-Supported MMHIs Versus Face-to-Face Interventions

A single item asked whether participants would prefer to use a peer-supported MMHI or a face-to-face peer-supported mental health intervention. A 5-point scale was used such that participants could either indicate no preference, a mild preference (somewhat prefer), or a strong preference (definitely prefer) for either MMHIs or face-to-face interventions.

#### Qualitative Variables

After participants were asked whether they would prefer to use a peer-supported MMHI or a face-to-face peer-supported mental health intervention, an open-ended question asked their reasoning behind their preference. At the end of the survey, another open-ended question asked whether there was anything else the participants wanted to share with the research team (see Multimedia Appendix 2 for a copy of each investigator-devised scale and items used for this study).

### Analytic Strategy

Quantitative data were analyzed using R version 4.3.1 [47]. In instances of missing item-level data, we prorated scores by averaging the available items. Before proration, 62 (11.4%) participant responses had some level of missingness across measures. After proration, 40 (7.3%) participant responses still contained some level of missingness across measures.

### Quantitative Analyses

To examine predictors of interest in MMHIs, we conducted ordinal logistic regression, designed for modeling an ordinal dependent variable [48], in a 2-step approach. In the first stage, we ran a comprehensive model of the complete list of 13 predictors: Hispanic ethnicity, the male gender, age, income, ethnic identity centrality, internet access, smartphone access, mental help–seeking attitudes, the level of depression symptoms, the level of social anxiety symptoms, computer attitudes, self-reported video game use, and experience with VR. In the second stage, we evaluated a parsimonious model that retained the predictors that were significant in the comprehensive model at *P*<.10, similar to the procedures used previously to examine predictors of response variables in digital interventions [49,50].

Given the bimodal distribution of preference for MMHIs versus face-to-face interventions responses, we dichotomized this outcome variable. To examine the predictors of MMHI versus face-to-face intervention preference, we conducted binary logistic regression using the same 2-stage approach described earlier.

To ensure the validity of the modeling approaches, we checked the assumptions of each comprehensive and parsimonious model. For ordinal logistic regression, we visually assessed the distribution of responses in our outcome variable (ie, interest in MMHIs) and noticed that responses indicating little to no interest (scores of 1 and 2) were highly associated with each other and responses indicating moderate-to-strong interest (scores of 4 and 5) were highly associated with each other. Although interest in MMHIs was relatively normally distributed, this raised concern for the validity of the proportional odds assumption in this data, which posits that the relationship (and, therefore, the odds ratio [OR] of coefficients) between each pair of outcome groups is the same [48]. As such, we collapsed the variable for interest in MMHIs into 3 categories: low interest (score of 1 or 2), neutral (score of 3), and high interest (score of 4 or 5). We checked for violations of the proportional odds assumption in the ordinal logistic model using the Brant test. For binary logistic regression, we visually checked for violations of the logit-linearity assumption between the outcome variable (ie, intervention preference) and continuous predictors. We assessed Cook’s distance and studentized residual plots for each comprehensive and parsimonious binary logistic regression to detect potential outliers. Five outliers were removed from the comprehensive binary logistic regression model. Lastly, we checked for multicollinearity in both parsimonious models, with a variance inflation factor of 4 or greater indicating high multicollinearity [51].

Results of a Wilcoxon rank sum test showed that Prolific participants reported a significantly lower mean MMHI interest compared to the non-Prolific sample (W=27258, *P*=.04). Therefore, we included *recruitment method* as a covariate in our model examining predictors of MMHI interest, which did not alter the direction or significance of results. We did not find a significant difference in MMHI versus face-to-face intervention preference with respect to recruitment method.

### Qualitative Analyses of Open-Ended Responses

We conducted an inductive content analysis [52] on open-ended responses explaining participant preference for MMHIs versus face-to-face interventions. Analysis followed all 8 guidelines set out by Cofie et al [53] for maintaining reflexivity and reliability using a qualitative-based measure of intercoder reliability (see Multimedia Appendix 3 for the checklist). Coding was performed using ATLAS.ti version 23.2.3.27778 for Windows [54]. First, authors FNR and RAB conducted an initial review of the data and generated a code framework. Second, all comments were coded by FNR and RAB, who subsequently discussed code groups and definitions and addressed points of contention. This step was repeated 3 times. The analytic process was recorded in research diaries, and no outstanding disagreements were observed after the discussions.

## Results

### Sample Characteristics

A total of 545 participants completed this study. The median survey completion duration was 8.9 minutes. A full sociodemographic breakdown of the sample assessed in this study is presented in Table 1.

**Table 1:** Characteristics of participants (N=545) at baseline.

Baseline characteristics		Values
**Sociodemographic factors**		
	Hispanic ethnicity, n (%)	161 (29.6)
	Age (years), mean (SD)	34.69 (12.75)
	Ethnic identity centrality, mean (SD)	3.04 (1.28)
	Access to the internet, n (%)	473 (86.8)
	Access to a smartphone, n (%)	490 (89.9)
**Gender, n (%)**		
	Male (reference)	261 (47.9)
	Female	265 (48.6)
	Genderqueer	2 (0.4)
	Nonbinary/nonconforming	14 (2.6)
	Unknown	3 (0.6)
**Mental health factors, mean (SD)**		
	Mental help–seeking attitudes	5.58 (1.19)
**Depression symptom severity, n (%)**		
	None	234 (42.9)
	Mild	160 (29.4)
	Moderate	92 (16.9)
	Moderately severe	33 (6.1)
	Severe	22 (4.0)
	Clinical level of social anxiety symptoms	184 (33.8)
**Technology factors, mean (SD)**		
	Computer attitudes	13.43 (5.94)
	Online video game use	3.14 (1.34)
	VR^a^ experience	2.11 (0.92)

^a^VR: virtual reality.

### Primary Analyses

#### General Interest in MMHIs

Regarding general interest in MMHIs, 192 (36.9%) of 520 participants reported low interest, 144 (27.7%) participants reported neutral interest, and 184 (35.4%) reported high interest. In the parsimonious model, older age, higher ethnic identity centrality, lack of internet access, more positive mental help–seeking attitudes, more online video game use, and more VR experience were associated with greater interest in MMHIs. Results of this parsimonious ordinal logistic model are presented in Table 2 (see Multimedia Appendix 4 for results of the comprehensive ordinal model).

**Table 2:** Predictors of general interest in peer-supported MMHIs^a^ in the parsimonious ordinal logistic regression model and predictors of preference for peer-supported metaverse versus face-to-face interventions in the parsimonious binary logistic regression model.

Predictors			Ordinal model						Binary model				
		OR^b^ (95% CI)		SE		*P* value		OR (95% CI)		SE		*P* value	
Hispanic			—^c^		—		—		1.81 (1.13-2.90)		0.24		.01
Male gender			—		—		—		0.43 (0.27-0.68)		0.23		<.001
Age			1.03 (1.02-1.05)		0.007		<.001		1.04 (1.02-1.05)		0.01		<.001
Ethnic identity centrality			1.44 (1.25-1.66)		0.07		<.001		1.28 (1.09-1.51)		0.08		.003
Internet access			0.50 (0.30-0.84)		0.26		.01		0.12 (0.02-0.40)		0.69		.002
Smartphone access			—		—		—		10.46 (2.87-50.71)		0.71		<.001
Mental help–seeking attitudes			1.22 (1.06-1.42)		0.08		.008		0.76 (0.62-0.92)		0.10		.005
**Depression symptoms^d^**													
	Mild	—		—		—		0.85 (0.51- 1.42)		0.26		.54	
	Moderate	—		—		—		0.60 (0.32- 1.12)		0.32		.11	
	Moderately severe	—		—		—		0.20 (0.07- 0.51)		0.49		.001	
	Severe	—		—		—		0.26 (0.08- 0.79)		0.58		.02	
Computer attitudes			—		—		—		1.05 (1.01-1.10)		0.02		.02
Video game use			1.26 (1.09-1.44)		0.07		.001		1.25 (1.05-1.48)		0.09		.01
VR^e^ experience			1.55 (1.28-1.90)		0.10		<.001		—		—		—

^a^MMHI: metaverse mental health intervention.

^b^OR: odds ratio.

^c^Not applicable.

^d^“No depression” was used as the reference group for all depression symptom variables.

^e^VR: virtual reality.

#### Preference for MMHIS Versus Face-to-Face Interventions

A total of 233 (42.8%) participants reported preference for MMHIs over face-to-face interventions, 223 (40.9%) participants reported preference for face-to-face interventions over MMHIs, and 64 (11.7%) participants indicated no preference. In the parsimonious model, Hispanic ethnicity, older age, higher ethnic identity centrality, smartphone access, more positive computer attitudes, and higher self-reported video game use were associated with greater odds of preference for MMHIs (versus face-to-face interventions). The male gender, internet access, more positive mental help–seeking attitudes, and higher levels of depression symptoms were associated with lower odds of preference for MMHIs (versus face-to-face interventions). Results of this parsimonious binary logistic model are presented in Table 2 (see Multimedia Appendix 4 for results of the comprehensive binary logistic model).

### Qualitative Analyses of Open-Ended Responses

We analyzed 483 comments elaborating on participant preference for peer-supported MMHIs versus peer-supported face-to-face mental health interventions. Analysis revealed 14 codes describing reasons for participants’ indicated preference: anonymity; social aversion; anxiety; comfort; ease of use and accessibility; ecological validity of social interaction; ecological validity of intervention; impersonal quality; aversion/distrust toward technology, the metaverse, or others in the metaverse; mental health attitudes; novelty/experience; privacy; immersion/engagement; and miscellaneous. An overview of the codes and their definitions by preference, as well as representative quotes for each, is presented in Multimedia Appendix 5.

Reasons related to anonymity or benefits of anonymity, such as reduced bias from others (133/483, 27.5%); an aversion to social, particularly face-to-face, interaction (38/483, 7.9%); greater ease of use and accessibility in the preferred intervention (35/483, 7.2%); anxiety, particularly social anxiety (28/483, 5.8%); and greater general comfort/greater comfort with negative feelings in the preferred intervention (26/483, 5.4%) tended to be endorsed by participants who indicated preference for MMHIs. Reasons related to poor ecological validity of social interaction in the nonpreferred intervention, such as lack of body language (99/483, 20.5%); poor ecological validity of the nonpreferred intervention itself, such as unrealistic graphics (75/483, 15.5%); aversion to the metaverse or distrust of affiliated companies or others in the metaverse social space (42/483, 8.7%); an “impersonal quality” of the nonpreferred intervention, often tied to perceived insincerity and overly distant social relations (31/483, 6.4%); and anticipated difficulty remaining engaged in the nonpreferred intervention (11/483, 2.3%) tended to be endorsed by respondents who preferred face-to-face interventions. Reasons related to mental health attitudes (28/483, 5.8%), privacy (19/483, 3.9%), and miscellaneous points (46/483, 9.5%) were endorsed approximately equally across preferences. The novelty of MMHIs/a lack of experience with MMHIs or mental health interventions (21/483, 4.3%) was most cited by those who expressed no preference.

## Discussion

### Principal Findings

Our quantitative analysis revealed that older age, higher ethnic identity centrality, lack of internet access, more positive mental help–seeking attitudes, more online video game use, and more VR experience were associated with greater odds of more interest in MMHIs. Hispanic ethnicity, older age, higher ethnic identity centrality, smartphone access, more positive computer attitudes, and higher self-reported video game use were associated with greater odds of preference for MMHIs (versus face-to-face interventions). The male gender, internet access, more positive mental help–seeking attitudes, and higher levels of depression symptoms were associated with lower odds of preference for MMHIs (versus face-to-face interventions). Our qualitative analysis revealed 14 themes related to participants’ reasons for why they prefer MMHIs versus face-to-face interventions. Among respondents who preferred MMHIs, reasons cited touched upon themes related to anonymity, social aversion, ease of use and accessibility, anxiety, and comfort. Among those who preferred face-to-face interventions, reasons touched upon ecological validity of social interaction, ecological validity of intervention, aversion/distrust toward technology, impersonal quality, and immersion/engagement. Mental health attitudes, privacy, and miscellaneous reasons were endorsed equally between preferences, and novelty was most cited by those who expressed no preference.

### Comparison to Prior Work

Regarding sociodemographic variables, our findings suggest that older adults may be more likely to report higher interest in MMHIs and find MMHIs more attractive than face-to-face interventions, assuaging potential concerns about the accessibility of MMHIs to these individuals [55]. Furthermore, internet access was found to be associated with a lower likelihood of reporting higher interest in MMHIs and of preferring MMHIs to face-to-face interventions. It may be that individuals without internet access are less familiar with the metaverse or online social environments and may find the idea novel and more appealing. Smartphone access was associated with greater odds of preference for MMHIs over face-to-face interventions. Smartphones are often cheaper and more easily accessed than a stable home internet connection, suggesting that those without smartphone access may be particularly averse to or unfamiliar with MMHIs or their type, such as DMHIs. Greater ethnic identity centrality also appeared to be associated with a greater likelihood of reporting higher interest in MMHIs, as well as a greater likelihood of preference for MMHIs versus face-to-face interventions. Qualitative responses from metaverse preferers often seemed to reflect an expectation of reduced bias and discrimination for their race or appearance in MMHIs as opposed to face-to-face interventions, possibly due to lower salience of identity in the metaverse. Hispanic ethnicity was associated with greater odds of preferring MMHIs as opposed to face-to-face interventions but not associated with interest in MMHIs. It is possible that Hispanics are more likely to prefer MMHIs to face-to-face interventions but not more likely to be interested in using a mental health intervention than non-Hispanics.

Regarding our examination of mental health factors, the greater likelihood of those with more positive attitudes toward mental help–seeking to be more interested in MMHIs might suggest that those who view mental help–seeking more positively may be more likely to view mental health interventions more positively in general. However, the association between more negative attitudes toward mental help–seeking and greater preference for MMHIs versus face-to-face interventions may indicate potential for peer-supported MMHIs to reach out to individuals who otherwise view mental health interventions unfavorably. This was echoed in the qualitative data by respondents who felt that discussing their mental health issues would be easier or less embarrassing in an MMHI than in a face-to-face intervention. However, we also found that participants with moderately severe or severe depression symptoms had greater odds of preferring face-to-face interventions to MMHIs than those with no depression symptoms. This may suggest that peer-supported MMHIs (which are low intensity) may not currently be a more attractive mental health intervention option than face-to-face intervention options for those struggling with more severe depression. Finally, we did not find a relationship between clinical levels of social anxiety symptoms and attitudes toward MMHIs, but our qualitative analysis did reveal a theme of anxiety, wherein comments tended to suggest that the respondents anticipated feeling less nervous in an MMHI than in a face-to-face intervention, often due to their anonymity. The findings regarding the relationship between anxiety and attitudes toward peer-supported MMHIs necessitate further study to elucidate the relationships between these factors.

Finally, the positive association between all 3 technology factors and at least 1 of the outcome variables, combined with the importance of the perceived “realness” of MMHIs and social interactions within them, as revealed in the qualitative analysis, suggests that the digital platform and technological novelty of MMHIs may factor into participant attitudes toward these interventions. However, only online video game use was significant in both ordinal and binary logistic regression models, possibly due to greater conceptual overlap of the videogame-like nature of current MMHIs. The promotion of MMHIs to technologically inclined individuals should be encouraged to connect help-seeking individuals with mental health services they are likely to be interested in. However, our findings also indicate that low ratings on certain technology factors, such as experience with VR, could serve as a barrier to willingness to use an MMHI. Furthermore, given the inconsistent associations between the technological factors measured and our dependent variables, there are likely other technological factors we did not assess that are associated with attitudes toward MMHIs. Attempting to replicate these findings by investigating lack of experience with technology and other technology factors as potential barriers to seeking MMHIs should be a direction of future research.

### Strengths and Limitations

To the best of our knowledge, this is the first study to identify factors associated with general interest in peer-supported MMHIs, as well as preference for peer-supported MMHIs versus peer-supported face-to-face mental health interventions. Our mixed methods approach was able to capture complementary quantitative and qualitative data that can offer a more comprehensive assessment (than either approach alone) of factors influencing participants’ attitudes toward MMHIs. However, we noted some limitations of this study. First, we examined a limited number of factors that could influence attitudes toward peer-supported MMHIs. We tried to mitigate this limitation by conducting a thorough review of the literature to identify candidate predictors across a variety of domains (sociodemographic, mental health, and technology). Nonetheless, future work would benefit from investigating other factors. Second, we examined predictive factors of attitudes toward peer-supported MMHIs in a general sample. Assessing these factors in a clinical sample may return different findings that help explain the similarly low level of interest in MMHIs as with face-to-face interventions for individuals with clinical depression or social anxiety. However, we did collect depression and social anxiety symptom data and observed that our sample had representation in each of the symptom severity categories assessed on the measures. Third, it was not always clear what factors an individual participant considered when reporting attitudes. A participant may or may not have considered the metaverse setting, the peer support element, the mental health emphasis, or a number of other factors. Although we sought to contextualize reported preferences using qualitative data, more work is needed to deepen the field’s understanding of the most salient aspects of peer-supported MMHIs that attract or deter participants.

### Future Directions

Future work could benefit from assessing the role of other attitudes (eg, attitudes toward companies hosting MMHIs) or technology factors that may be more directly relevant to MMHI use (eg, confidence/competence with technology, number of hours using related technology). We also encourage future research on the acceptability and feasibility of MMHIs with diverse samples to ensure the validity and generalizability of these findings.

### Conclusion

This study assessed attitudes toward peer-supported mental health interventions delivered via the metaverse. By identifying sociodemographic, mental health, and technology factors associated with attitudes toward peer-supported MMHIs, our findings represent the first venture into identifying the individuals and populations who may be especially willing to use peer-supported MMHIs or for whom MMHIs may be more attractive than face-to-face interventions (eg, older adults, those with more negative mental help–seeking attitudes). The results of this study serve to inform the future development and outreach plans of MMHIs by identifying populations that may be particularly likely or unlikely to be interested in MMHIs. If replicated, this information could potentially be used to optimize treatment outreach to interested populations, especially those who may not seek mental help otherwise (eg, by increasing the quantity and promotion of technical support, including help documents, to encourage MMHI use by older adults), or to change aspects of MMHIs to better appeal to disinterested populations. Future work is encouraged to confirm the salience of the factors identified in this study to attitudes toward MMHIs and to assess predictive factors of outcomes when using an MMHI.

## Appendix

Multimedia Appendix 1 Checklist for Reporting Results of Internet E-Surveys (CHERRIES).

Multimedia Appendix 2 Investigator-devised scales and items used for this study.

Multimedia Appendix 3 Checklist of recommendations for reporting intercoder reliability in qualitative research from Cofie et al [53].

Multimedia Appendix 4 Results of comprehensive logistic models.

Multimedia Appendix 5 Themes from qualitative analysis of open-ended responses.

## Abbreviations

DMHI: digital mental health intervention
MHSAS: Mental Help Seeking Attitudes Scale
Mini-SPIN: Mini-Social Phobia Inventory
MMHI: metaverse mental health intervention
OR: odds ratio
USC: University of Southern California
VR: virtual reality

## Data Availability

The data sets generated and analyzed during this study are available from the corresponding author upon reasonable request.
