# Supplementary material for: Assessing Predictive Factors of Attitudes Toward Peer-Supported Mental Health Interventions in the Metaverse: Mixed Methods Study
Source: JMIR XR Spat Comput. 2024 Aug 22;1:e57990. doi: 10.2196/57990 (PMC13179108; doi:10.2196/57990)
Supplement: Multimedia Appendix 2 [file xr_v1i1e57990_app2.docx]

**Multimedia Appendix 2.** Investigator-devised scales and items used for this study.

**Level of Ethnic Identity Centrality**

How important is your racial or ethnic background to your identity as a whole?

1: Not at all important

2: Slightly important

3: Moderately important

4: Very important

5: Extremely important

**Internet Use Habits**

When you have accessed the Internet via a computer, smartphone or other device, how often do you engage in the following activities?

1. Education-related tasks (e.g. attending online courses, completing assignments, etc.)
2. Work-related tasks (e.g. sending business e-mails, videoconferencing, completing work-related projects, etc.)
3. Social communication (e.g. sending personal e-mails, using voice communication software, using social media, etc.)
4. Browsing the Web for any personal purpose (e.g. reading news, online shopping, personal investing, etc.)
5. Listening to music or watching movies, television shows, or videos
6. Playing online video games
7. Independent work (e.g. cloud programming projects, cloud art projects, etc.)

1: Never

2: Rarely

3: Sometimes

4: Often

5: Very Often

**Level of Experience with Virtual Reality**

Do you have any prior experience using virtual reality (VR) technology?

(Virtual reality is a computer-generated 3D environment that a user can explore and interact with. Virtual reality is often accessed through virtual reality headsets such as the one shown. [Image])

1: No experience

2: A little experience

3: Some experience

4: A lot of experience

**Level of Interest in Peer-Supported MMHIs**

If money was not a concern, how willing would you be overall to use a **metaverse** mental health peer support program such as Innerworld?

1: Not at all willing

2: A little willing

3: Moderately willing

4: Very willing

5. Extremely willing

**Preference for Peer-Supported MMHIs vs. Face-to-Face Interventions**

If money was not a concern for either, would you prefer to use a **metaverse** mental health peer support program where you attend groups as an anonymous avatar OR a **face-to-face** mental health peer support program where you attend groups in-person?

1: Definitely prefer metaverse

2: Somewhat prefer metaverse

3: No preference

4: Somewhat prefer face-to-face

5. Definitely prefer face-to-face
