# Supplementary material for: Assessing Predictive Factors of Attitudes Toward Peer-Supported Mental Health Interventions in the Metaverse: Mixed Methods Study
Source: JMIR XR Spat Comput. 2024 Aug 22;1:e57990. doi: 10.2196/57990 (PMC13179108; doi:10.2196/57990)
Supplement: Multimedia Appendix 3 [file xr_v1i1e57990_app3.docx]

**Multimedia Appendix 3.** Checklist of recommendations for reporting intercoder reliability in qualitative research from Cofie et al. (2022).

| Aspects of Intercoder Reliability | Present | | Justification (if “no” selected) |
| --- | --- | --- | --- |
| There was a minimum of two coders. | **Yes** | No |  |
| At least one coder was more removed from data collection (to address bias). | **Yes** | No |  |
| At least one coder had expertise and previous experience with coding qualitative data. | **Yes** | No |  |
| If there were multiple participant groups, a minimum of two researchers (coders) coded transcripts from each participant group. | Yes | No | N/A: this study did not have multiple experimental/observational groups. |
| The coders used the same framework for analysis (e.g., inductive, deductive, abductive). | **Yes** | No |  |
| Coders focused on shared meaning of *codes through dialogue and consensus. | **Yes** | No |  |
| Another coder with expertise in qualitative methods was consulted to resolve outstanding conflicts. | Yes | No | N/A: unneeded. No conflicts were outstanding after final discussion between coders. |
| Coder consensus resulted in a codebook** that was applied when coding the remaining transcripts. | **Yes** | No |  |

* The code names do not have to be identical, but the meaning of the codes must be the same.

**In inductive and abductive analyses, coding can be an iterative process; therefore, new codes may be added to the codebook until code saturation is reached.
