# Supplementary material for: Assessing Predictive Factors of Attitudes Toward Peer-Supported Mental Health Interventions in the Metaverse: Mixed Methods Study
Source: JMIR XR Spat Comput. 2024 Aug 22;1:e57990. doi: 10.2196/57990 (PMC13179108; doi:10.2196/57990)
Supplement: Multimedia Appendix 4 [file xr_v1i1e57990_app4.docx]

**Multimedia Appendix 4.** Results of comprehensive logistic models.

|  | Comprehensive Ordinal Model | | | | Comprehensive Binary Model | | | |
| --- | --- | --- | --- | --- | --- | --- | --- | --- |
| Predictors/Covariates | OR^a^ | SE | 95% CI | *P* | OR^a^ | SE | 95% CI | *P* |
| Prolific participant | 0.48 | 0.22 | [0.31,0.73] | <.001 | 0.45 | 0.29 | [0.25,0.78] | .005 |
| Hispanic | 1.37 | 0.20 | [0.93,2.03] | .11 | 1.80 | 0.24 | [1.13,2.90] | .01 |
| Male gender | 0.99 | 0.19 | [0.69,1.45] | .99 | 0.43 | 0.23 | [0.27,0.68] | <.001 |
| Age | 1.04 | 0.008 | [1.02,1.05] | <.001 | 1.04 | 0.01 | [1.02,1.06] | <.001 |
| Ethnicity centrality | 1.44 | 0.07 | [1.25,1.67] | <.001 | 1.29 | 0.08 | [1.09,1.52] | .002 |
| Internet access | 0.48 | 0.42 | [0.21,1.08] | .08 | 0.11 | 0.69 | [0.02,0.39] | .002 |
| Smartphone access | 1.02 | 0.48 | [0.40,2.63] | .97 | 10.76 | 0.71 | [2.94,52.30] | <.001 |
| MH^b^ seeking attitudes | 1.24 | 0.08 | [1.06,1.45] | .006 | 0.75 | 0.10 | [0.62,0.92] | .005 |
| Dep.^c^ Mild | 1.07 | 0.23 | [0.68,1.68] | .76 | 0.81 | 0.27 | [0.47,1.38] | .44 |
| Dep. ^c^ Moderate | 1.14 | 0.2 | [0.66,1.98] | .64 | 0.54 | 0.35 | [0.27,1.08] | .08 |
| Dep. ^c^ Moderately Severe | 1.11 | 0.40 | [0.51,2.45] | .79 | 0.18 | 0.53 | [0.06,0.48] | <.001 |
| Dep. ^c^ Severe | 0.50 | 0.48 | [0.19,1.29] | .16 | 0.22 | 0.63 | [0.06,0.74] | .02 |
| SA^d^ symptoms | 1.44 | 0.22 | [0.93,2.22] | .10 | 1.21 | 0.27 | [0.71,2.06] | .49 |
| Computer attitudes |  |  |  |  | 1.05 | 0.02 | [1.01,1.10] | .02 |
| Video game use | 1.25 | 0.07 | [1.08,1.44] | .003 | 1.24 | 0.09 | [1.04,1.48] | .02 |
| VR^e^ Experience | 1.55 | 0.10 | [1.27,1.90] | <.001 | 1.03 | 0.12 | [0.82,1.31] | .78 |

*Note*. Predictors of interest in peer-supported metaverse mental health interventions in the comprehensive ordinal logistic regression model and predictors of preference for peer-supported metaverse vs. face-to-face interventions in the comprehensive logistic regression model.

^a^ Odds ratio.

^b^ Mental help.

^c^ Measure of depressive symptoms. No depression was used as the reference group for all depression symptom variables.

^d^ Clinical level of social anxiety.

^e^ Virtual reality.
