# Supplementary material for: Assessing Predictive Factors of Attitudes Toward Peer-Supported Mental Health Interventions in the Metaverse: Mixed Methods Study
Source: JMIR XR Spat Comput. 2024 Aug 22;1:e57990. doi: 10.2196/57990 (PMC13179108; doi:10.2196/57990)
Supplement: Multimedia Appendix 5 [file xr_v1i1e57990_app5.docx]

**Multimedia Appendix 5.** Themes from qualitative analysis of open-ended responses.

| Code (definition) | # Metaverse Preference | # Face-to-Face Preference | # No Preference | Quote from Metaverse Preference | Quote from Face-to-Face Preference | Quote from No Preference |
| --- | --- | --- | --- | --- | --- | --- |
| Anonymity  (Mention of anonymity or perceived benefits of anonymity) | 108 | 20 | 5 | “being in the metaverse is a little appealing to remain anon” | “i would rather see who the real person is, not their avatar” | “I feel I would benefit from both, but I do like the anonymous nature of the metaverse option” |
| Social Aversion  (Mention of aversion to social interaction with other people) | 36 | 1 | 1 | “Because I don't like being around people so I would prefer the anonymous Avatar” | “the less that people know the better for me because i like my privacy and would feel embarrassed with many people even if it is annoynomous” | “i'm not social in either. i have no preference since it's no difference for me” |
| Ease of use and accessibility  (Mention of an intervention’s ease of access and/or ease of use) | 30 | 2 | 3 | “The metaverse option would be significantly more convenient and immediate rather than structured face-to-face programs.” | i like having to leave my house consistently” | “I don't take issue with talking face to face with people, I just don't have a vehicle.” |
| Anxiety  (Mention of anxiety or an anxious disorder) | 22 | 4 | 2 | “I have social anxiety, so I think it would make me less anxious to use an avatar to talk to someone vs face to face.” | “I feel like face-to-face therapy helps me more due to my main problem being social anxiety.” | “I think there's merit to both. The metaverse can ease tension and anxiety of having to meet and talk to people face to face, however in-person has the upside of giving more real connections.” |
| Comfort  (Mention of greater general comfort/greater comfort with negative feelings in preferred intervention) | 20 | 4 | 2 | “i would feel more safe” | “I am not as comfortable in a VR setting as in person.” | “I am comfortable in either position” |
| Ecological validity of social interaction  (Mention of the quality, validity or verisimilitude of social interaction within the intervention) | 4 | 89 | 6 | “So I don't read into the therapist's body language wrong, I would prefer the metaverse where I can also be anonymous.” | “face to face interaction is more real. better for humans I think.” | “It wouldn't matter if it was face to face or through the metaverse. I feel like the conversation is the most important part rather than being in person or not.” |
| Ecological validity of intervention  (Mention of the quality, validity or verisimilitude of an intervention itself) | 1 | 67 | 7 | “Metaverse would seem more realistic.” | “Hard to take things seriously when I am just an anonymous avatar in a video game world.” | “I think as long as the program was effective, it wouldn’t matter me which format it was in.” |
| Aversion/distrust towards technology, the metaverse, or others in the metaverse  (Mention of aversion to technology in general, the metaverse or affiliated companies, or skepticism regarding the honesty of others in the social space) | 0 | 42 | 0 | n/a | “I want nothing to do with the Metaverse. I won’t use it.”  “I think it's too easy for people behind screens to boldly misuse this program, so I'd prefer in person.” | n/a |
| Impersonal quality  (Mention of the general level of personalness/intimacy of the intervention) | 2 | 29 | 0 | “Seems more personable” | “Face to face just seems more personal to me. I worry I’m vr I just wouldn’t get that personal feeling.”.” | n/a |
| Mental health attitudes  (Mention of mental help-seeking behavior or stigma with regards to help-seeking behavior) | 11 | 14 | 3 | “Many people feel uncomfortable or shy when dealing with mental health issues, and seeking help or advice from strangers or professionals may find it difficult to speak up.” | “I feel like things such as mental health help is best doing it in person. There is more human contact and I feel like they will understand you better.” | “Either option sounds interesting, however, I would avoid both as I believe attending groups for mental health solutions to be extremely unhelpful.” |
| Novelty/experience  (Mention of one’s desire to try new things or one’s familiarity with an intervention or mental health interventions) | 5 | 5 | 11 | “I like to try new things” | “More experience with face to face” | “I have no preference because I have no experience with a metaverse program.” |
| Privacy  (Mention of one’s desire to maintain privacy, including data privacy) | 9 | 9 | 1 | “for privacy would be the main reason i would prefer the metaverse” | “I don't trust that data sent through the metaverse is safe or private.” | n/a |
| Immersion/engagement  (Mention of one’s anticipated engagement/focus in an intervention) | 3 | 8 | 0 | “I think that this method would allow me to forget about the things that make me feel insecure and just focus on the experience in front of me.” | “I would prefer face-to-face because I would feel more engaged in the activity by attending in person. I would also feel less distracted and could devote more of my attention this way.” | n/a |
| Miscellaneous | 20 | 21 | 5 | “Because I like the distant universe.” | “Its perfect ot me” | “Sometimes face to face is fine. I dont mind metaverse if someone else has paid for the best setup for me.” |
| No Reason Specified | 3 | 2 | 17 | “I think it’s interesting” | “Face to face is better” | “I wouldn’t do it either way.” |
